# Novel macromolecules derived from coumarin: synthesis and antioxidant activity

**DOI:** 10.1038/srep11825

**Published:** 2015-07-02

**Authors:** Ahmed A. Al-Amiery, Yasameen K. Al-Majedy, Abdul Amir H. Kadhum, Abu Bakar Mohamad

**Affiliations:** 1 Department of Chemical and Process Engineering, Universiti Kebangsaan Malaysia (UKM), Bangi, Selangor 43000, Malaysia

## Abstract

The rational design of 4-hydroxycoumarins with tailor-made antioxidant activities is required nowadays due to the wide variety of pharmacologically significant, structurally interesting of coumarins and researcher orientation toward green chemistry and natural products. A simple and unique coumarins have been achieved by reaction of 4-hydroxycoumarin with aromatic aldehyde accompanied with the creation of a macromolecules have 2-aminothiazolidin-4-one. The molecular structures of the compounds were characterized by the Fourier transformation infrared and Nuclear magnetic resonance spectroscopies, in addition to CHN analysis. The scavenging abilities of new compounds against stable DPPH radical (DPPH•) and hydrogen peroxide were done and the results show that the compounds exhibited high antioxidant activates.

The legend of coumarin was started in 1820 when Vogel extracted it from Tonka beans. Coumarin is a natural product that occurring in plants and it molecule chemically consists of a benzene ring fused to a lactone ring^1^. Coumarins are far reaching in plants including numerous vegetables, flavors, soil grown foods, and therapeutic plants, most of them are not destructive to humans in the amounts present in consumable plants^2^. Coumarins have variety important biological activities such as anti-inflammatory, anti-oxidant^3^, anti-viral^4^, anti-microbial^5^ and anti-cancer^6^. Recently coumarins have been indicated to increase central nervous system activity^7^. Coumarins were utilized as flavoring agents in, toothpastes, foods, detergents, tobaccos, alcoholic and beverages^8^. In industry, coumarin derivatives were commonly used as laser dyes due to their emission properties^9^. Free radicals are molecules or atoms (free particles or ions) having no less than one unpaired electron; subsequently, they are very active with an extensive variety of other molecules. They are always created and kept up in balance in biological systems through metabolic processes, furthermore they assume important roles in a variety of typical biochemical capacities, for example, cell signaling, apoptosis, gene expression, ion transport, and pathological processes^10^,^11^. Our studies started from the design of novel coumarins with enhanced antioxidant activities. We approach to increasing of antioxidant activities based on the long conjugated system in the novel synthesized coumarins.

## Results and Discussion

### Chemistry

**Figure 1:**
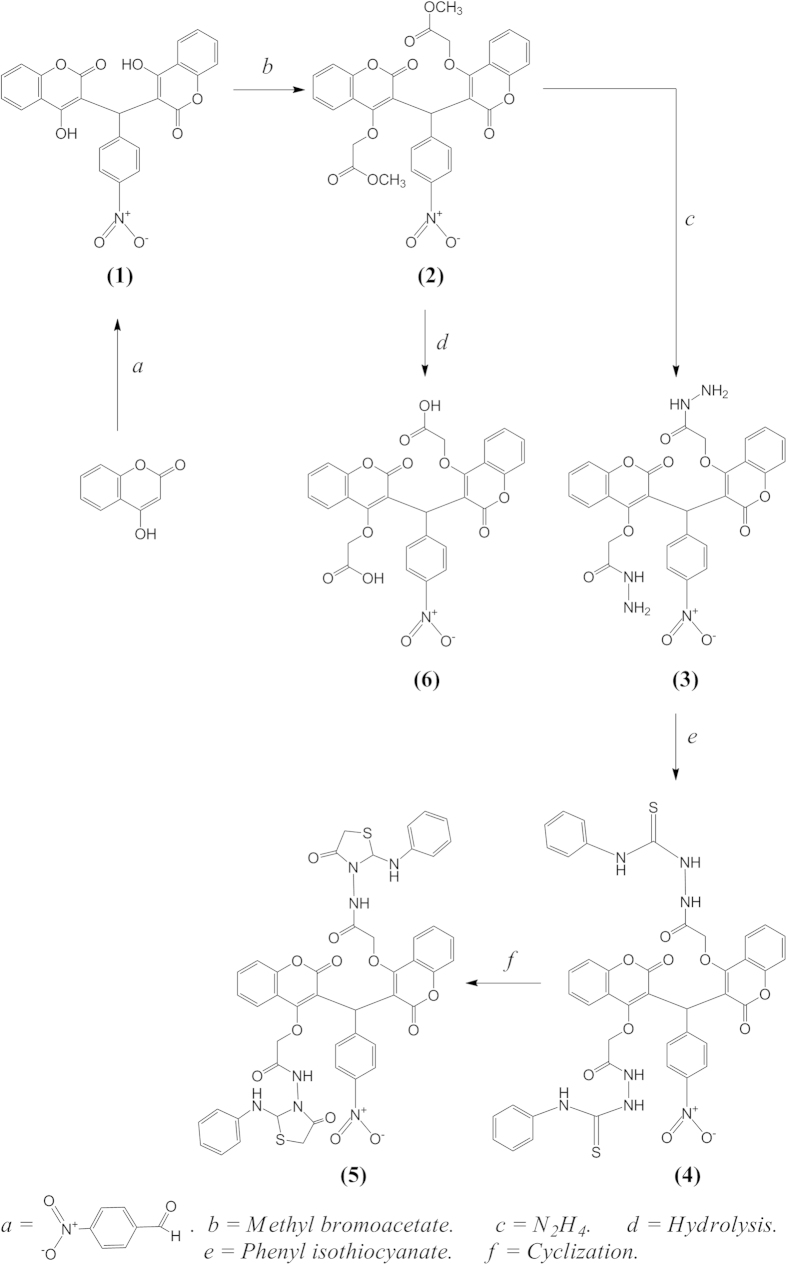
The synthesis of new antioxidant compounds.

The reaction sequence for the synthesis of new antioxidant compounds (**1–6**) is outlined in Fig. 1, 3,3′-((4-nitrophenyl)methylene)bis(4-hydroxy-2H-chromen-2-one) (**1**) was prepared according to the literature method that mentioned referenced^12^,^13^ by condensation reaction of 4-hydroxycoumarin with 4-nitrobenzaldehyde in the presence of glacial acetic acid. The structure of (**1**) was inferred from the analytical and spectral data. Thus, IR spectra showed characteristic absorption bands at 3314 cm^−1^ (OH), 1683 cm^−1^ (lactone C=O) and 3082 cm^−1^ (CH-aromatic). The ^1^H-NMR spectrum of (**1**) exhibited singlet at δ 6.10 (1H, s, C-H). The synthesis of dimethyl 2,2′-((3,3′-((4-nitrophenyl)methylene)bis(2-oxo-2H-chromene-4,3-diyl))bis(oxy))diacetate (**2**) was obtain by reflux of methyl bromoacetate with 3,3′-((4-nitrophenyl)methylene)bis(4-hydroxy-2H-chromen-2-one) (**1**) in the presence of anhydrous potassium carbonate and acetone. Hydrazinolysis of **2** with hydrazine hydrate produced 2,2′-((3,3′-((4-nitrophenyl)methylene)bis(2-oxo-2H-chromene-4,3-diyl))bis(oxy))di(acetohydrazide) **(3)** in good yield. The FT-IR spectrum of (**3**) showed absorption bands in the 3281 and, 3213 cm^−1^ (hydrazide, NH-NH_2_), 1710 cm^−1^ (lactonic -C=O carbonyl stretching) and 1614 cm^−1^ (amide -C=O carbonyl stretching). The ^1^H-NMR spectrum exhibited a singlet due to the -CO-NH-NH_2_ proton at δ 8.65 ppm. Reflux of **(3)** with phenyl isothiocyanate in ethanol gave 4-phenyl-1-(7-hydroxy-2-oxo-2H-chromen-4acetyl-)-thiosemicarbazide (**4**), the IR spectrum of (**4**) showed characteristic bands at 3289 and 3216 cm^−1^ (NH), 1782.2 cm^−1^ (C=O, lactone), 1652 cm^−1^ (C=O, amide). The ^1^H-NMR spectrum showed the absence of NH_2_ protons. 2,2′-((3,3′-((4-nitrophenyl)methylene)bis(2-oxo-2H-chromene-4,3-diyl))bis(oxy))bis(N-(4-oxo-2-(phenylamino)thiazolidin-3-yl)acetamide) (**5**) was obtain by reaction of (**4**) with methyl bromoacetate in presence of anhydrous NaOAc for 9 hr, The IR spectrum of (**5**) showed characteristic bands at 3303 and 3141 cm^−1^ (NH), 1698 (C=O, lactone). 2,2′-((3,3′-((4-nitrophenyl)methylene)bis(2-oxo-2H-chromene-4,3-diyl))bis(oxy))diacetic acid (**6**) was obtained from hydrolyses of (**2**) by concentration hydrochloric acid, the IR spectrum of (**6**) showed characteristic bands at 3421 cm^−1^ (OH), 1733 cm^−1^ (C=O, acid), 1654 cm^−1^ (C=O, lactone) and the ^1^H-NMR spectrum showed the presence of OH proton at 10.9 ppm.

### Pharmacology

**Figure 2:**
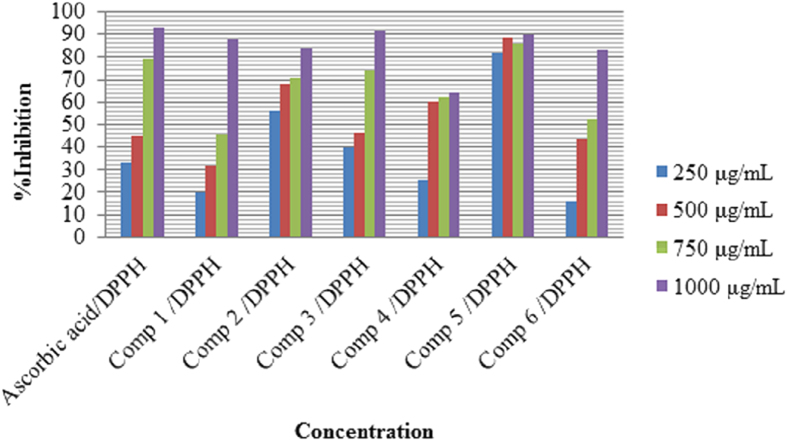
Effect of compound (1–6) toward 1,1-diphenyl-2-picrilhydrazyl (DPPH).

**Figure 3:**
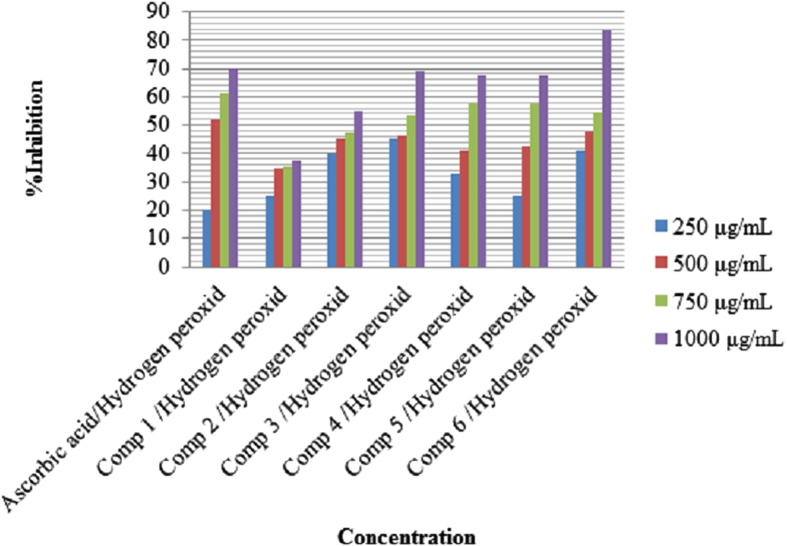
Effect of compound (1–6) toward hydrogen peroxide.

The role of antioxidant is to remove free radicals. One important mechanism through which this is achieved is by donating hydrogen to free radicals in its reduction to nonreactive species^14^. Addition of hydrogen would remove the odd electron feature which is responsible for radical reactivity. Free radicals have been a subject of significant interest among scientists in the past decade. Their broad range of effects in biological systems has drawn the attention of many workers^15^. It has been proven that free radicals play an important role in the pathogenesis of certain diseases and aging^16^. Many synthetic antioxidant components have shown toxic and/or mutagenic effects, and therefore attention has been paid to naturally occurring antioxidants. Compounds (**1–6**) were screened for *in vitro* antioxidant activity using DPPH (2,2-diphenyl-1-picrylhydrazyl) radical and H_2_O_2_ (hydrogen peroxide). They show good antioxidant activity (Figs 2 and 3). The hydrogen-donating activity, measured using 2,2-diphenyl-1-picrylhydrazyl and hydrogen peroxide radicals as hydrogen acceptor, showed that significant association could be found between the concentration of the new synthesized molecule and the percentage of inhibition. Through 2,2-diphenyl-1-picrylhydrazyl and hydrogen peroxide test, compounds (**1**–**6**) have been shown to reduce the stable radical. According to Fig. 2 the synthesized compounds (**1**), (**3**) and (**5**), possessing 88%, 92%, and 89.6% DPPH radical scavenging activity. These compounds contain 3,4-dihydroxyphenyl ring. Compounds with these substituents are expected to possess antioxidant activity^17^, since hydrogen donation leads to formation of a stable quinoid-structure. It has been reported that two hydroxyl groups are important for antioxidant activity^18^,^19^. The scavenging effect increased with the increasing concentrations of test compounds. In DPPH method, the maximum scavenging activity was 92% at a concentration 1000 μg/mL for compound (**3**) and the minimum scavenging activity was 16% at a concentration of 250 μg/mL for compound (**6**). The antioxidant activities of (**1–6**) were done using DPPH (2,2-diphenyl-1-picrylhydrazyl) radical and H_2_O_2_ (hydrogen peroxide) scavenging methods. Ascorbic acid was used as the standard. Figures 2 and 3, indicates the antioxidant activities of (**1–6**).

### Postulated Mechanisms for (1) as Antioxidant

**Figure 4:**
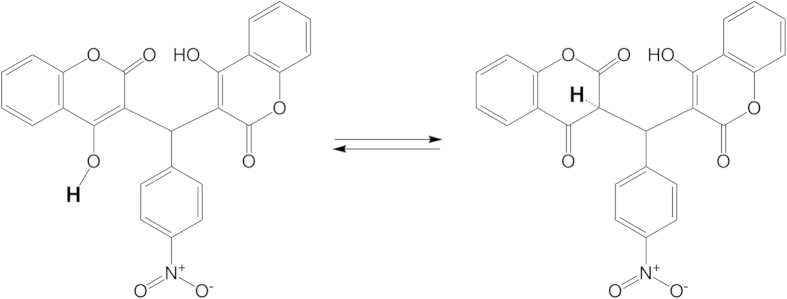
The postulated mechanism for compound (1) as antioxidant.

Suggested mechanisms for the reaction of (**1**) as an antioxidant as shown in Fig. 4, that depends on the hydroxyl hydrogen atom the **bolded** one, whereas this atom was under the influence resonance effect. The tautomer’s play an important role in releasing of hydrogen, it can been seen from Fig. 4 that the benzyl carbon atom is under withdrawing of three groups, first one is the aryl group that has nitro group in para position that has the highest electron with drawing and the second, third groups are the carbonyls. The resonance effect of carbon-benzylic one- makes the release of hydrogen as a free radical easier.

## Methodology

### Chemistry

The chemicals used during synthesis were supplied by Sigma-Aldrich (Selangor, Malaysia). The IR spectra were obtained on a Nicolet 6700 FT-IR spectrophotometer (Thermo Nicolet Corp., Madison, WI, USA), and the values are expressed in cm^−1^. Nuclear magnetic resonance (NMR) spectra were recorded using an AVANCE III 600 MHz spectrometer (Bruker, Billerica, MA, USA), using DMSO as an internal standard and the values are expressed in δ ppm.

#### *3,3*′*-((4-nitrophenyl)methylene)bis(4-hydroxy-2H-chromen-2-one)* (**1**)

Refluxation of 4-hydroxycoumarin (3.24 g, 20 mmol) with 4-nitrobenzaldehyde (1.51 g, 10 mmol) in 50 mL of glacial acetic acid for 300 min, than yellow crystals appear. After cooling the yellow crystals was filtered and recrystallized from acetonitrile. Yield 70%, m.p 232–233 °C; ^1^H-NMR (CDCl_3_): δ 6.10 (1H, s, C-H); 7.51–7.43 (2H, m, Ar-H); 7.464−7.492 (2H, m, Ar-H); 7.881 (H, d, *J* = 7.8 Hz, Ar-H); 8.09 (H, d, *J* = 7.6 Hz, Ar-H); 11.03 (H, s, O-H); ^13^C-NMR (CDCl_3_): 161.8, 160.2, 152.9, 149.3, 143.4, 134.7, 125.1, 127.7, 124.5, 122.9, 118.6, 115.8, 100.0, 35.6; IR (KBr): 3314 (O-H), 3082 (C-H Aromatic), 1683 (C=O), 1614, 1544, 1514, 1176, 762 cm^−1^. Theoretical Calculation for C_25_H_15_NO_8_: C 65.65%, H 3.31%, N 3.06%; Experimental C 65.27%, H 3.05% N 2.94%.

#### *Dimethyl 2,2*′*-((3,3*′*-((4-nitrophenyl)methylene)bis(2-oxo-2H-chromene-4,3-diyl))bis(oxy))diacetate* (**2**)

A suspension of (**1**), (5.118 g, 11.2 mmol) with potassium carbonate (3.408 g, 24.7 mmol) in acetone (100 mL) was refluxed for 30 min then methyl bromoacetate (4.256 g, 28 mmol) was added over a 5 min period and the resulting solution was refluxed for further 2000 min. Cooling, filtering and evaporated. The solid was recrystallized from methanol; yield 45%; m.p 211–214 °C; ^1^H-NMR (CDCl_3_): δ 3.58 (3H, s, CH_3_); δ 4.82 (2H, s, CH_2_); δ 6.01 (1H, s, C-H); 7.324−7.277 (2H, m, Ar-H); δ 7.63−7.43 (2H, s, Ar-H); 7.824 (H, d, *J* = 7.8 Hz, Ar-H); 8.11 (H, d, *J* = 7.6 Hz, Ar-H); ^13^C-NMR (CDCl_3_): 166.3, 161.2, 160.3, 152.8, 151.1, 141.1, 127.1, 127.0, 125.4, 124.4, 122.9, 115.6, 111.2, 94.9, 61.3, 50.5, 36.3; IR: 3081 cm^−1^ (C-H, Aromatic), 2961 cm^−1^ (C-H, Aliphatic), 1754 cm^−1^ (C=O, Esteric), 1715 cm^−1^ (C=O, Lactonic) 1552 cm^−1^ (C=C, Aromatic); Theoretical Calculation for C_31_H_23_NO_12_: C 61.90%, H 3.85%, N 2.33%; Experimental: C 61.63% H 3.62%, N 2.26.

#### *2,2*′*-((3,3*′*-((4-nitrophenyl)methylene)bis(2-oxo-2H-chromene-4,3-diyl))bis(oxy))di(acetohydrazide)* (**3**)

A solution of (**2**) (6.01 g, 10 mmol) in ethanol 25 mL was refluxed with hydrazine hydrate (10.12 g, 20 mmol) for 240 min. The solid mass separated out and recrystallized using ethanol, yield 50%; m.p 237–240 °C; ^1^H-NMR (CDCl_3_): δ 2.05 (2H, s, NH_2_); δ 4.61 (s, 2H, CH_2_); δ 6.01 (1H, s, C-H); δ 7.59−7.43, (2H, m, Ar-H); 7.68–7.61 (2H, s, Ar-H); 7.90 (H, d, *J* = 7.8 Hz, Ar-H); 8.03 (H, d, *J* = 7.6 Hz, Ar-H); ^13^C-NMR (CDCl_3_):: 170.4, 165.9, 160.4, 150.3, 144.3, 122, 126.4, 123.9, 125.1, 129.3, 116.2, 115.8, 95.4, 67.5, 36.9. IR: 3281.1, 3213 cm^−1^ (N-H), 3061.5 cm^−1^ (C-H, Aromatic), 2903.0 cm^−1^ (C-H, Aliphatic), 1710.1 cm^−1^ (C=O, Lacton), 1614. 7 cm^−1^ (C=O, Amide); Theoretical Calculation for C_29_H_23_N_5_O_12_: C 54.98%, H 3.66%, N 11.05%. Experimental: C 54.57% H 3.72%, N 11.33%.

#### *2,2*′*-(2,2*′*-((3,3*′*-((4-nitrophenyl)methylene)bis(2-oxo-2H-chromene-4,3-diyl))bis(oxy))bis(acetyl))bis(N-phenylhydrazinecarbothioamide)* (**4**)

A mixture of (**3**) (6.01 g, 10 mmol) and phenyl isothiocyanate (2.702 g, 20 mmol) in 50 ml of absolute ethanol was refluxed for 180 min. The crude product thus obtained was filtered and recrystallized from ethanol. yield 48.5%, m.p 273–274 °C; ^1^H-NMR (CDCl_3_): δ 7.01–7.11, (2H, m, Ar-H), δ 7.42−7.39, (2H, m, Ar-H), δ 7.69−7.61 (2H, s, Ar-H); 7.89 (H, d, *J* = 7.8 Hz, Ar-H); 8.22 (H, d, *J* = 7.6 Hz, Ar-H) δ 6.01 (1H, s, CH), δ 4.65 (2H, s, CH_2_), δ 2.1 (1H, s, NH), δ 4.11, (1H, s, NH); δ 8.02 (1H, s, NH); ^13^C-NMR (CDCl_3_):: 37.5, 67.4, 95.8, 115.1, 117.0, 123.8, 124.1, 125.6, 126.9, 127.1, 127.4, 127.9, 128.6, 129.1, 137.4, 143.6, 151.5, 153.1, 161.5, 162.3, 165.9, 179.2. IR: 3289.1, 3216 cm^−1^ (N-H), 3051.0 cm^−1^ (C-H, Aromatic) 2901 cm^−1^ (C-H, Aliphatic), 1728.2 cm^−1^ (C=O, Lacton), 1652.1 cm^−1^ (C=O, Amide); Theoretical Calculation for C_43_H_33_N_7_O_10_S_2_: C 59.23%, H 3.81%, N 11.25%. Experimental: C 58.9% H 3.5%, N 10.95%.

#### *2,2*′*-((3,3*′*-((4-nitrophenyl)methylene)bis(2-oxo-2H-chromene-4,3-diyl))bis(oxy))bis(N-(4-oxo-2-(phenylamino)thiazolidin-3-yl)acetamide)* (**5**)

A mixture of (**4**) (8.72 g, 10 mmol) and methyl bromoacetate (3.04 g, 20 mmol) were refluxed in 30 ml of absolute ethanol in the presence of anhydrous NaOAc (3.28 g, 40 mmol) for 9 hr. the reaction mixture was cooled, diluted with water and allowed to stand overnight .The solid precipitated was washed with water, dried and recrystallized from ethanol. yield 56.4%, m.p. 288–289 °C ; ^1^H-NMR (CDCl_3_): ^1^H-NMR (CDCl_3_): 8.24 (H, d, *J* = 7.6 Hz, Ar-H), δ 8.11 (1H, s, NH), 7.78 (H, d, *J* = 7.8 Hz, Ar-H); δ 7.62−7.54 (2H, m, Ar-H), δ 7.34−7.23 (2H, m, Ar-H), δ 6.01 (2H, s, CH_2_), δ 4.62 (2H, s, CH_2_), δ 3.91, 3.85 (2H, d, CH_2_), δ 4.1 (1H, s, NH); ^13^C-NMR (CDCl_3_):: 34.2, 37.6, 65.7, 83.0, 97.1, 114.6, 115.5, 116.8, 121.2, 123.6, 124.0, 125.5, 126.2, 128.9, 144.3, 147.2, 151.1, 152.3, 160.7, 162.2, 165.7, 168.5. IR: 3303.5, 3141.3 cm^−1^ (N-H), 3052.1 cm^−1^ (C-H, Aromatic), 2903.0 cm^−1^ (C-H, Aliphatic), 1709.3 cm^−1^ (C=O, Lacton), 1698.8 cm^−1^ (C=O, Amide); Theoretical Calculation for C_47_H_37_N_7_O_12_S_2_: C 59.05%, H 3.90%, N 10.26%. Experimental: C 58.90% H 3.53, N 10.65%.

#### *2,2*′*-((3,3*′*-((4-nitrophenyl)methylene)bis(2-oxo-2H-chromene-4,3-diyl))bis(oxy))diacetic acid* (**6**).

A mixture of (**2**) (4.657 g, 7.75 mmol) in distilled water (50 ml) and concentration hydrochloric acid (37%, 5 ml) was refluxed for 300 min. On cooling ,the solid was filtered off, washed with cold ethanol and water and then dried for 4 days in vacuum at 50 °C, yield 35%; m.p. 241–243. ^1^H-NMR (CDCl_3_): δ 4.55 (2H, s, CH_2_); δ 6.01 (1H, s, C-H); 7.49−7.42 (2H, m, Ar-H); δ 7.62, −7.52 (2H, s, Ar-H); 7.81 (H, d, *J* = 7.8 Hz, Ar-H); 8.14 (H, d, *J* = 7.6 Hz, Ar-H); ^13^C-NMR (CDCl_3_): 170.8, 162.5, 161.6, 152.4, 144.3, 128.3, 126.3, 125.3, 123.9, 116.2, 114.5, 96.1, 63.8, 37.1. IR: 3421.9 cm^−1^ (OH), 3055.1 cm^−1^ (C-H, Aromatic), 2878.2 cm^−1^ (C-H, Aliphatic), 1733.3 cm^−1^ (C=O, Acid), 1654.3 cm^−1^ (C=O, Lacton); Theoretical Calculation for C_29_H_19_NO_12_: C 60.74%, H 3.34%, N 2.44%. Experimental: C 59.88% H 3.01%, N 2.13%.

### Biochemistry

#### DPPH

DPPH [(2,2-diphenyl-1-picrylhydrazyl)] Radical Scavenging Activity 0.1 mL of the synthesized compounds (**1–6**) at concentration of 250, 500, 750 and 1000 μg/mL (or ascorbic acid) was mixed with 1 mL of 0.2 mM DPPH and dissolved in methanol than after the mixture was incubated in the dark for 20 min at 28 °C^20^. The scavenging activity was determined by measuring the absorbance at 517 nm using the UV-VIS spectrophotometer. The percentage of DPPH radical scavenger was calculated utilizing Equation 1.
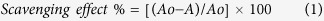
A_o_ = Control absorbance; A = Tested absorbance

#### H_2_O_2_ (Hydrogen Peroxide) Scavenging Activity

H_2_O_2_ was prepared in phosphate buffer at power of hydrogen equal to 7.4. The synthesized compounds with concentration of (250, 500, 750 and 1000) μg/mL (or ascorbic acid) were added to a hydrogen peroxide solution (0.6 mL, 40 mM). Absorbance of H_2_O_2_ at 230 nm was determined after 10 min^17^. against a blank solution containing phosphate buffer without hydrogen peroxide. H_2_O_2_ scavenging activity was then calculated using Equation 1.

## Conclusions

It is evident that coumarin and coumarin-related compounds are a plentiful source of potential drugs candidate in relation to their safety and efficacy. New coumarin derivatives as a macromolecules have been synthesized and characterized using various spectroscopic methods and elemental analysis technique. The antioxidant compounds (**1–6**) were tested and they exhibited significant activities. In addition, compounds (**1, 3**) and (**5**) were also found to be superior to ascorbic acid with respect to their antioxidant activities.

## Additional Information

**How to cite this article**: Al-Amiery, A. A. *et al*. Novel macromolecules derived from coumarin: synthesis and antioxidant activity. *Sci. Rep*. **5**, 11825; doi: 10.1038/srep11825 (2015).
